# Delayed hepatic rupture post ultrasound-guided percutaneous liver biopsy: A case report

**DOI:** 10.1097/MD.0000000000009955

**Published:** 2018-03-02

**Authors:** Jia-Yan Huang, Qiang Lu, Ji-Bin Liu

**Affiliations:** a Department of Ultrasound, West China Hospital of Sichuan University, Chengdu, China; b Department of Ultrasound/Radiology, Thomas Jefferson University, Philadelphia, PA.

**Keywords:** complications, hemorrhage, liver biopsy, liver rupture, ultrasound

## Abstract

**Rationale:**

Hemorrhage, one of complications after liver biopsy, is often identified immediately after the procedure while delayed liver rupture is relatively rare.

**Patient concerns:**

A 45-year-old woman was diagnosed with undetermined liver cirrhosis and abnormal liver function. To determine the etiology and severity of liver cirrhosis, ultrasound-guided liver biopsy was arranged. The patients did not complain any pain during the procedure. Ultrasound examination on postoperative day1 (POD 1) and MRI on POD 3 showed no evidence of hematoma and ascites. On POD 7, however, the patient was taken to the hospital with a sudden onset of pain in the right upper quadrant of the abdomen.

**Diagnoses:**

Contrast-enhanced computed tomography revealed liver rupture of right inferior segment of the liver with subcapsular hematoma.

**Interventions:**

Patient was treated with infusion of 2-unit red blood cell suspension, fluid and hemostatics.

**Outcomes:**

The vital signs of the patient were stabilized after the therapy. The follow-up ultrasound 1 month later showed a shrunken subcapsular hematoma measuring 4.2 × 2.1 cm at the right lobe.

**Lessons:**

Whenever a liver biopsy procedure is performed, the care should be taken to avoid puncturing those areas that may have liver incisure. Moreover, the patient need to rest for several days and to avoid heavy activities, which is one of the major risk factors for post-procedure bleeding.

## 1 Introduction

Percutaneous liver biopsy has been widely accepted as a standard care for evaluation of liver disease.^[1]^ The use of Imaging guidance for liver biopsy markedly reduces the incidence of procedure-related complications.^[2,3]^ Ultrasound, because of its real-time imaging and relative low cost, becomes first choice of modality and the most widely used method. However, due to the invasive nature of this procedure, complications are sometimes inevitable. Pain is the most common complication, followed by bleeding.^[4]^ Hemorrhage after liver biopsy is often identified immediately after the procedure while delayed liver rupture is relatively rare. We report a case of delayed liver rupture 1 week after ultrasound-guided liver biopsy.

## 2 Case report

**Figure 1:**
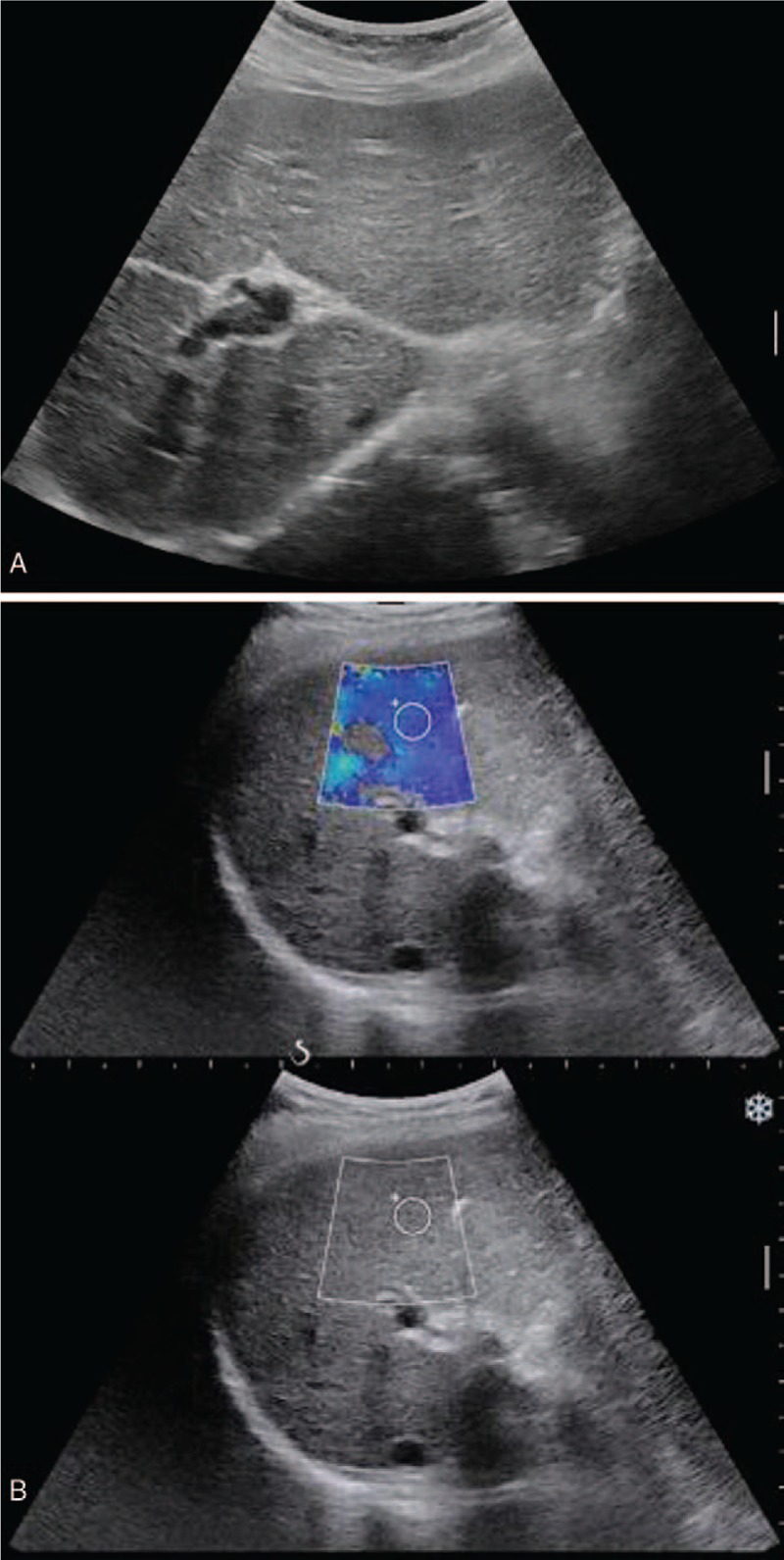
Ultrasound image of a 45-year-old woman with abnormal liver function. (A) Grayscale sonogram demonstrated coarse echotexture of the liver parenchyma and shrunken right lobe. (B) Cirrhosis was suspected with an elevated liver stiffness of 11 KPa measured by shear wave elastography.

**Figure 2:**
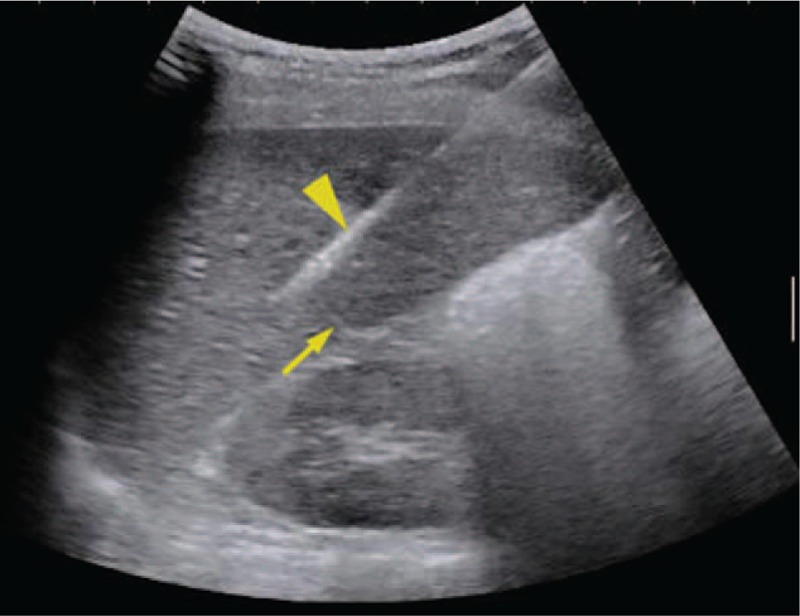
During the biopsy, a BARD automatic biopsy gun with 18G needle (arrow head) was applied under the real-time ultrasound guidance. An echogenic line thought to be capsule at the liver incisures (arrow) was punctured during the procedure.

**Figure 3:**
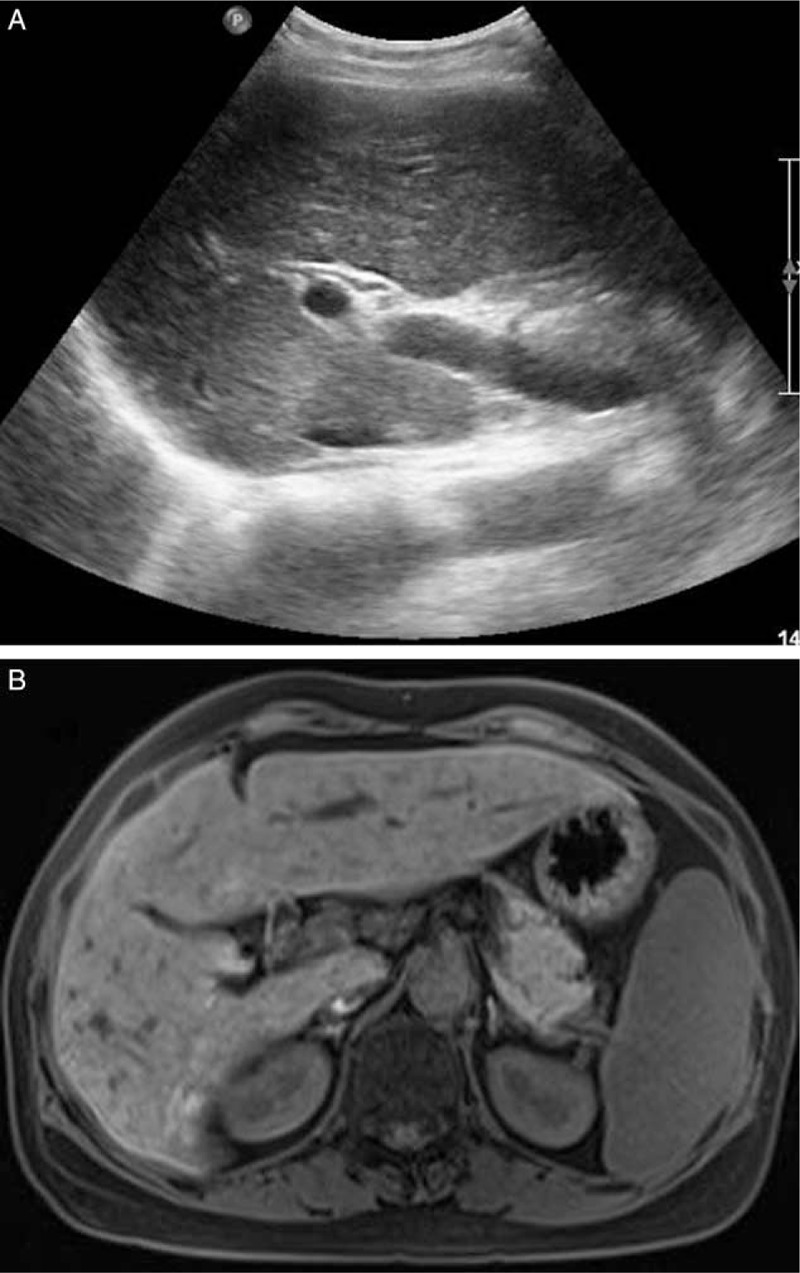
Ultrasound and MRI scan of the patient after the biopsy. The ultrasound exam on POD 1 (A) and MRI on POD 3 (B) show no sign of hematoma and ascites.

**Figure 4:**
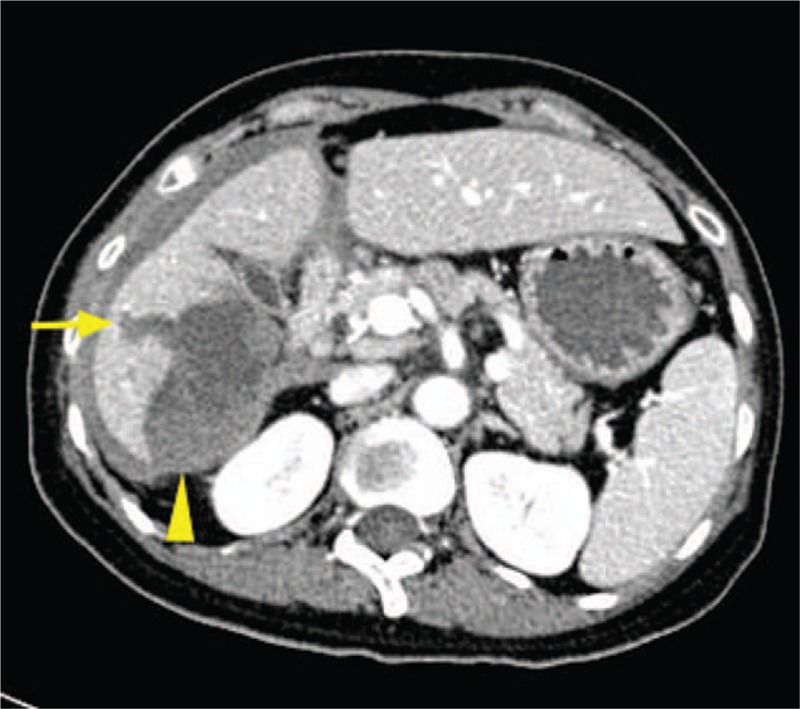
Contrast-enhanced computed tomography (CECT) at the Department of Emergency. CECT demonstrated liver rupture (arrow) at the right inferior segment of the liver with subcapsular hematoma (arrowhead).

**Figure 5:**
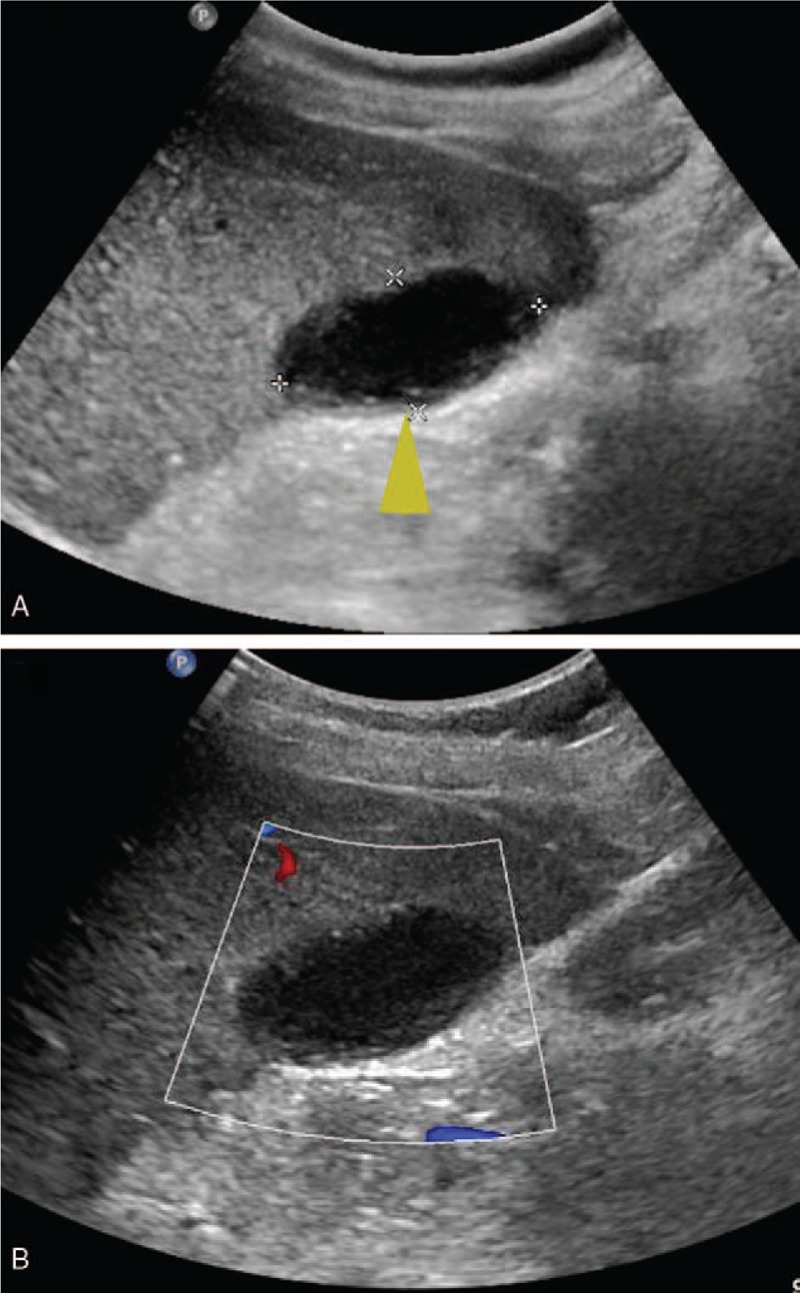
Follow-up ultrasound 1 month later. (A) Grayscale ultrasound illustrated an anechoic lesion measuring 4.2 × 2.1 cm at the right lobe (arrowhead). (B) The lesion was absent of Doppler signal on Color Doppler ultrasound, which was in accordance with subcapsular hematoma.

The Institutional Ethics Committee approved this retrospective study and waived written informed consent. A 45-year-old woman with undetermined liver cirrhosis and abnormal liver function was administrated in our hospital. Laboratory examination revealed AST = 65 IU/L, ALT = 86 IU/L, TB = 17.2 μmol/L, GGT = 361 IU/L, ALB = 43.8 g/L, PLT = 158 × 10^9^, PT = 10.4 s, and INR = 0.88. Preoperative ultrasound demonstrated coarse echotexture of liver parenchyma and shrunken right lobe. The value of liver stiffness was 11 kPa, which was measured by shear wave elastography (Aixplorer ultrasound [US] system; SuperSonic Imagine, Aix-en-Provence, France) with a convex broadband probe (SC6–1) (Fig. 1). To determine the etiology and severity of liver cirrhosis, ultrasound-guided liver biopsy was carried out with Aixplorer ultrasound system (SuperSonic Imagine) and a convex broadband transducer (SC6–1). After a careful transabdominal scanning of the liver, the segment VI of the right lobe was chosen for the target of biopsy. With the patient at left decubitus position, a BARD automatic biopsy gun with 18G needle was applied for core tissue sampling under real-time ultrasound guidance (Fig. 2). Two tissue core samples with 2 cm in length were obtained from the targeted area of the liver and the pathology proved to be primary biliary cirrhosis. The patients did not complain any pain during the procedure and was asked to rest on bed for 4 hours. Ultrasound examination on POD 1 and MRI on POD 3 showed no evidence of hematoma and ascites (Fig. 3). The patient was discharged on POD 5. On POD7, the patient was taken to the hospital with a sudden onset of pain in the right upper quadrant of the abdomen. Contrast-enhanced computed tomography revealed liver rupture at the right inferior segment of the liver with subcapsular hematoma (Fig. 4). The blood pressure was 100/67 mmHg with HBG of 62 g/L at admission. The vital signs of the patient were stabilized after conservative management by blood transfusion of 2 unit red blood cell suspension, fluid infusion, and hemostatics. The follow-up ultrasound 1 month later showed a shrunken subcapsular hematoma measuring 4.2 × 2.1 cm at the right lobe (Fig. 5).

## 3 Discussion

Liver biopsy acts as a final qualitative interpretation of the liver disease after a clinical history, physical examination, biochemical, serological, and imaging investigation have failed to elucidate a diagnosis.^[5]^ The use of imaging guidance during the biopsy procedure markedly reduces the incidence of procedure-related complications. Real-time ultrasound guidance is convincing, clinically beneficial, and relatively low cost,^[3,6]^ which was taken as the most widely used method for guiding biopsy.

Patients who require liver biopsy often have damaged liver function and abnormal coagulation status. Complications of biopsy, such as pain, minor bleeding, and hypotension is not uncommon.^[7]^ A variety of other miscellaneous complications, including hemothorax, biliary peritonitis, intrahepatic arteriovenous fistula formation, and even death were also reported.^[8–11]^ Liver rupture is a severe complication after liver biopsy, which may lead to death.^[12]^ In this case, two conceivable factors may contribute to the delayed liver rupture with hemorrhage. Firstly, more than one site of liver capsule was punctured due to anatomic variation. Liver incisure at the visceral surface is not uncommon. The echogenic line (Fig. 2) at the puncture site was thought to be the hepatic capsule at the liver notch, which was punctured during the procedure. Secondly, exertion when doing housework may be an inducement. Patients with advanced chronic liver disease were also regarded as a potential reason leading to serious bleeding after liver biopsy.^[13]^

Although liver rupture after percutaneous liver biopsy is rare, the care should be taken to avoid puncturing those areas that may have liver incisure. Moreover, the patient need to rest for several days and to avoid heavy activities, which is one of the major risk factors for post-procedure bleeding.
